# Establishing the Need for Anticipatory Symptom Guidance and Networked Models of Disease in Adaptive Family Management Among Children With Medical Complexity: Qualitative Study

**DOI:** 10.2196/52454

**Published:** 2023-11-23

**Authors:** Jessica Keim-Malpass, Christopher Lunsford, Lisa C Letzkus, Eleanore Scheer, Rupa S Valdez

**Affiliations:** 1 Division of Pediatric Hematology-Oncology Department of Pediatrics University of Virginia School of Medicine Charlottesville, VA United States; 2 Department of Physical Medicine and Rehabilitation Duke University School of Medicine Durham, NC United States; 3 Division of Developmental Pediatrics Department of Pediatrics University of Virginia School of Medicine Charlottesville, VA United States; 4 Department of Systems and Information Engineering University of Virginia School of Engineering and Applied Sciences Charlottesville, VA United States; 5 Department of Public Health Sciences University of Virginia School of Medicine Charlottesville, VA United States

**Keywords:** anticipatory symptom management, children with medical complexity, social network, qualitative, self-management, care coordination, precision health, disease progression models, disability, networked models of disease, social networking, web-based network, web-based networks, social networks, symptom management, family management, coordinated care, precision, pediatric, pediatrics, child, children, caregiver, caregivers, social support, interview, interviews, family, informal care, caregiving, conceptual, grounded theory, constructivist, situational analysis, model, mobile phone

## Abstract

**Background:**

Caregivers of children with medical complexity navigate complex family management tasks for their child both in the hospital and home-based setting. The roles and relationships of members of their social network and the dynamic evolution of these family management tasks have been underexamined.

**Objective:**

The purpose of this study was to explore the structures and processes of family management among caregivers of children with medical complexity, with a focus on the underlying dynamic nature of family management practices and the role of members of their social network.

**Methods:**

This study used a qualitative approach to interview caregivers of children with medical complexity and members of their social network. Caregivers of children with medical complexity were recruited through an academic Children’s Hospital Complex Care Clinic in the mid-Atlantic region and interviewed over a period of 1 to 3 days. Responses were analyzed using constructivist grounded theory and situational analysis to construct a new conceptual model. Only caregiver responses are reported here.

**Results:**

In total, 20 caregivers were included in this analysis. Caregiver perspectives revealed the contextual processes that allowed for practices of family management within the setting of rapidly evolving symptoms and health concerns. The dynamic and adaptive nature of this process is a key underlying action supporting this novel conceptual model. The central themes underpinning the adaptive family management model include symptom cues, ongoing surveillance, information gathering, and acute on chronic health concerns. The model also highlights facilitators and threats to successful family management among children with medical complexity and the networked relationship among the structures and processes.

**Conclusions:**

The adaptive family management model provides a basis for further quantitative operationalization and study. Previously described self- or family management frameworks do not account for the underlying dynamic nature of the disease trajectory and the developmental stage progression of the child or adolescent, and our work extends existing work. For future work, there is a defined role for technology-enhanced personalized approaches to home-based monitoring. Due to the disparities caregivers and the children in this population already experience, technology-enhanced approaches must be built alongside key stakeholders with an equity orientation to technology co-development.

**International Registered Report Identifier (IRRID):**

RR2-10.2196/14810

## Introduction

Caregivers of children with medical complexity are often tasked with delivering substantial medical and health care activities in home- and community-based settings while also managing activities of daily living [1]. Children with medical complexity encompass a heterogenous group defined across several domains of care including family-identified health care service needs, chronic and serious conditions, functional limitations, and high health care use [2,3]. Further, children with medical complexity often require technology dependence to deliver vital nutrition and oxygen therapies that require consistent nursing-level care [4]. Because of the high-intensity care work, caregivers of children with medical complexity are at increased risk for financial and social hardships along with deleterious effects on their own physical and mental health and well-being [5,6]. These risks and consequences are highly dynamic and situational, often dependent on the stability and complexity of the child [6,7].

Much of the care delivered for children with medical complexity occurs in the home managed by the primary caregivers and can be framed by the conceptual terms of self-management or, more broadly, family management [4,8]. Family management practices include both direct care provision (eg, medication management, wound dressing changes, management of gastrostomy tube feeds, symptom management, surveillance, and assessment) and the coordination of care, financial and care coverage navigation, and information-seeking activities that support future care activities [4,9]. Caregivers must learn and adapt to integrate treatment recommendations and care plans from a variety of types of health care providers (eg, primary care, multiple specialists, behavioral, occupational or physical or speech therapy, and palliative or hospice) [10]. They must also navigate complex financial and insurance coverage pathways as many children with medical complexity can qualify for Medicaid based on functional assessment and severity of illnesses while also sometimes retaining private insurance [11,12]. Further, some children are eligible for Medicaid waivers or other state-based systems to cover care received in home- and community-based settings, but coverage, eligibility, and provision of these services can vary widely [11].

There are several gaps in our understanding of family management processes among children with medical complexity. First, the ways that caregivers engage and navigate family management processes in the context of their own social networks (which can include other family members, friends, community members, members of the care team, and members from web-based communities) is a much-needed area of inquiry [9,13]. Social network members connected to the caregiver and children with medical complexity engage in care processes in highly variable ways, and their involvement often requires additional layers of communication and coordination [14,15].

Another gap in our knowledge of family self-management in the care of children with medical complexity is the longitudinal evolution of these processes. Family management is a dynamic process with contextually driven therapeutic plans, symptom experiences, and financial or insurance coverage landscapes that often evolve with the child. Further, children grow within their own unique developmental trajectory, meaning that family management strategies for a 2-month-old will be different than a 2-year-old, which will be different for a school-age child and adolescent, even when it is the same child. Comorbidities and severity of illness also change over time, and it is incredibly difficult to prognosticate the underlying trajectory of illnesses [16]. Finally, the caregivers’ social network composition also evolves over time, as does the underlying nature of the relationships that bond the social network. The purpose of this study was to explore the structures and processes of family management among caregivers of children with medical complexity, with a focus on the underlying dynamic nature of family management practices and the role of members of their social network. One of the central goals of this work was to develop a conceptual model for future equity-oriented, technology-enabled interventions that can facilitate longitudinal or life-course family management practices among caregivers of children with medical complexity and their social networks.

## Methods

### Design and Participants

This study used a constructivist grounded theory methodological approach that focuses on structures, processes, and knowledge construction to create a new phenomenon [17-19]. This analysis is part of a larger qualitative assessment of social networks among children with medical complexity [9]. Caregivers were recruited through purposive sampling via a mid-Atlantic academic Children’s Hospital Complex Care Clinic: anyone in direct primary care of a child with medical complexity (defined as occupying the same household as the child). Caregivers also had to be 18 years or older to participate in the study. The criteria for identifying a child with medical complexity were established by the Center of Excellence on Quality of Care Measures for Children With Complex Needs. They characterize children with medical complexity as individuals younger than 21 months who have chronic conditions affecting 2 or more body systems, necessitating health care resources beyond what is typical for most children, and requiring ongoing treatment to manage their condition [20].

As part of the overarching study, caregivers were asked to identify members of their social network who were contacted and consented to perform individual interviews. The social network interviews were read for the overall context of this paper but were not explicitly analyzed or reported on for this analysis and are reported elsewhere [9]. Thus, the caregiver interviews were the focus of this paper. Demographic and clinical characteristics of the participants and their children were assessed in a web-based process to ensure maximum diversity sampling across a full range of child complexity domains and participant characteristics. Typically, the caregiver interviews lasted from 40 to 75 minutes. The interviews were conducted in a cross-sectional manner, but we used approaches in the interview process to better understand the evolution and changes in both the child’s own medical complexity and family management practices.

The interviews took place from October 2019 through March 2021 as the COVID-19 pandemic was unfolding, so we had to be flexible to the ongoing changes in human subject’s research participation during that time. The team already had approval to conduct the interviews digitally, but there were delays associated with the general workforce disruptions during COVID-19. We also modified our interview guide to account for perceptions of family management prior to the interruptions of the COVID-19 pandemic and developed a specific secondary interview for COVID-19–specific family management considerations and experiences. Participants had the option of either completing the interview in 1 session or completing it over the course of 2-3 days. All COVID-19–specific interviews happened on a day separate from the primary interviews and were read for context but were not included in the analysis of this work specifically. Interviews were conducted over the telephone and were audio recorded and transcribed verbatim.

### Ethical Considerations

This work was approved by the institutional review board (SBS 2182). Verbal informed consent was obtained for each study participant and for each identified social network member who participated. All participants were 18 years or older. Privacy and confidentiality were maintained by making the interview transcripts anonymous and linked by code. Participants were compensated with a US $40 gift card following the conclusion of the interviews.

### Analysis

**Figure 1:**
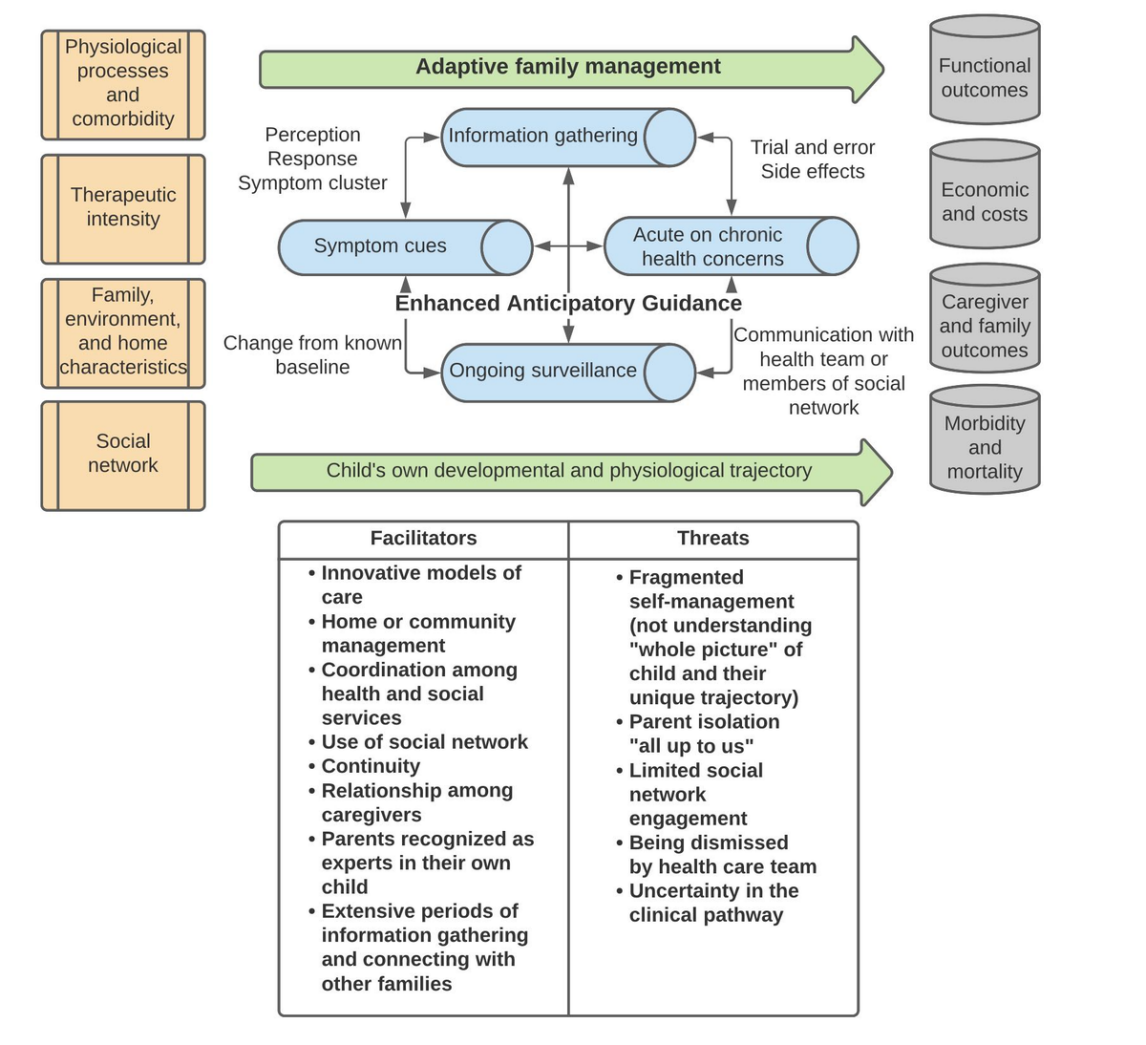
Adaptive family management conceptual framework to support enhanced anticipatory symptom guidance and networked models of disease.

Analysis was guided using the iterative process of constructivist grounded theory using the analytic strategy of situational analysis as described by Clarke [19,21]. The first phase of analysis involved reading the transcript and writing short memos and maps describing the contextual pieces of the interview and general perceptions about the overall actors (child, members of the family, and social networks), structures, processes, and relationships between the elements. The situational maps used the collective experience of family management in the context of having a child with medical complexity as the unit of analysis, and the maps were used to amplify open-coding structures. The next phases involved transforming the open codes to categories that encompassed several codes. Axial coding then placed the more salient categories into a conceptual structure with connections to define relationships. In the final stage, theoretical coding organized the axial codes into a coherent structure, where subsequent data findings were further auditioned as being central to the explanatory matrix as context, conditions, actions or interactions, or consequences. The explanatory matrix that best fits the story of the data was developed as Figure 1. This process allows for a final conceptual model that can be viewed with elements related to context, central actions (themes), central relationships, and outcomes. We also highlighted facilitators and threats to the relationships, actions, and themes that either aid or hinder functional family management of children with medical complexity. Trustworthiness and attention to rigor were addressed in the following ways: documenting key study design and analytic decisions, reflexivity practices involving open memoing and reflecting on prior and ongoing assumptions, and independent review of the final conceptual model by all coauthors [22].

## Results

### Sample Characteristics

Interviews from 20 caregiver participants were included in this analysis, and sample characteristics can be found in Table 1. The mean age of the sample was 35 (SD 7.9) years, with the majority of participants identifying as a woman (n=18, 90%) and White (n=17, 85%). Most (n=16, 80%) of the participants are able to access the internet both in the home and on their phone, and most (n=17, 85%) own a smartphone. In total, 40% (n=8) of the participants in this sample had marginal or limited health literacy. Medical conditions of the child along with reliance on technology can also be found in Table 1.

**Table 1:** Participant and child characteristics.

Participant characteristic (n=20)		Values
Age (years), mean (SD)		34.9 (7.9)
**Age, n (%)**		
	Age specified	18 (90)
	Age not specified	2 (10)
**Gender identity, n (%)**		
	Woman	18 (90)
	Man	2 (10)
**Own cell phone, n (%)**		
	Yes	18 (90)
	No	0 (0)
	Not specified	2 (10)
**Own smartphone, n (%)**		
	Yes	17 (85)
	No	1 (5)
	Not specified	2 (10)
**Highest level of education, n (%)**		
	Less than high school	1 (5)
	High school graduate or General Education Diploma Test	3 (15)
	Some college	9(45)
	2-year college degree	1 (5)
	4-year college degree	4 (20)
	Not specified	2 (10)
**Race, n (%)**		
	Black	2 (10)
	White	17 (85)
	Not specified	1 (5)
**Health literacy, n (%)**		
	Adequate	10 (50)
	Marginal or limited	8 (40)
	Not specified	2 (10)
**Access internet, n (%)**		
	Home and phone	16 (80)
	Phone only	2 (10)
	Not specified	2 (10)
**Medical conditions of child (not mutually exclusive), n (%)**		
	Prematurity	5 (25)
	Chronic lung disease	5 (25)
	Cerebral palsy	8 (40)
	Epilepsy	6 (30)
	Brain tumor	1 (5)
	Congenital heart disease	3 (15)
	Feeding difficulty or failure to thrive	3 (15)
	Congenital anomaly or genetic syndrome	7 (35)
	Behavioral or mental health	7 (35)
	Endocrine	1 (5)
**Technology dependence of child (not mutually exclusive), n (%)**		
	Home oxygen requirement	3 (15)
	Gastrostomy tube	3 (15)

### Central Action Supporting Adaptive Family Management

Caregiver perspectives revealed the contextual processes that allowed for family management practices responsive to rapidly evolving symptoms and health concerns. The evolution of disease manifestations, the complexity of co-occurring symptoms, and the growth of the child’s own developmental and physiological trajectory contribute to families constantly having to adjust to “new normals” both in terms of surveillance or monitoring and also new therapeutic strategies. Underlying medical complexity shapes the experiences of navigation and adjustment for families, as one caregiver described:

She was born with two conditions, and I was under the assumption that we would only have to deal with two conditions for life, and it seems like, as time has went by, she’s collected more, so I’m sure she’ll collect some more as we go.

Participant 20

Self-management processes for the family had to routinely evolve and adapt to the underlying changes in both the developmental and physiological conditions of the child. Further, factors that influence family management practices are not static, meaning that the level of social network and community support, well-being of the family, and intensity or place of care are dynamic processes that influence family management outcomes. Hence, the adaptive nature of this process is a key underlying action supporting this novel conceptual model. The central themes supporting the adaptive family management model include symptom cues, ongoing surveillance, information gathering, and acute on chronic health concerns (Figure 1). The model also highlights facilitators and threats to successful family management among children with medical complexity (Figure 1).

### Symptom Cues

Families of children with medical complexity undergo constant vigilance to routinely assess for subtle changes in their child’s status that could indicate a change from their baseline or a larger clinical issue. The theme symptom cues (Textbox 1) coincides with the notion that the caregiver is always the expert in the care of their child, and they are unfortunately often dismissed by members of the medical community. One participant describes it in the following way:

The second I know of is because of her nosebleeds. They [medical professionals] panic that it wouldn’t stop, and then I kept tellin’ them what to do. They wouldn’t listen to me, and she went into seizures. She kept having seizures every few minutes, so it would begin and then stop, begin and then stop, begin and then stop.

Participant 5

**Qualitative exemplars of the theme “symptom cues.”**

- “I look at her. I see if she looks like she’s having issues breathing like if it’s harder for her to breathe. And if so or if I see she’s doing something out of normal like when I see something that’s like ‘okay that’s not her.’” [Participant 1]
- “Oh, whenever the temperature and stuff—whenever she’s all of a sudden—she just quits doing anything. She just gets real—in her face she has—her color changes. Her lips, the color changes. She turns almost like a sheet, white. I just monitor her color. You can tell. Plus, she starts crying at times.” [Participant 4]
- “Do we need to increase the dose? Do we need to decrease the dose? You can’t do those things quickly. If you need to do an increased dose, it may be stepped up over the course of weeks or months even. We’ve done it before where it was over the period of eight weeks that we increased a particular medication.” [Participant 6]
- “He has an eating schedule to follow, but we just go by his cues. We could take him off of his ventilator just depending on how he’s acting. It’s not like set in stone he comes off at this time and goes back on at this time or anything like that.” [Participant 8]
- “Because I can typically just tell by her mood and her behavior. I know she’s about to get sick when she gets really quiet, when she doesn’t interact with us as much or isn’t as easily excitable. It’s harder to make her smile, or her appetite might decrease. There’s definitely cues that she gives us that are pretty easy to pick up on.” [Participant 9]
- “I usually just monitor her behaviors. If her behavior starts changing, that usually means that there’s a seizure coming on.” [Participant 16]

One participant describes it also in the following way:

They [medical professionals] will dismiss the concerns of the parents. I have learned that I don’t want to work with those doctors because they are the experts in their field and I am the expert in my child. If I have a concern, I want to be heard.

Participant 6

Because of the nature of their child’s medical complexity, caregivers must assess these cues through patterns that would not be apparent to others. As a caregiver describes:

Where she’s nonverbal, we have to kind of read her actions because she can’t tell us. Like if she has a headache, she’ll take her hand and hit her head and let us know that she has a headache.

Participant 15

The process of monitoring for early, subtle changes before apparent clinical signs are present was a uniform experience shared among participants. Personalized approaches to assessing for subtle symptom cues differed among participants, and most strategies for accomplishing this type of assessment were based on constant monitoring vigilance.

### Ongoing Surveillance

Related to the theme of symptom cues is the theme of ongoing surveillance that extends symptom cues by establishing processes for both routine and symptom-specific monitoring of their child (Textbox 2). As previously described, caregivers are constantly searching for small, subtle changes within their children and have established processes and structures for ensuring appropriate surveillance of their child’s changing needs. They also have numerous routine parameters (eg, daily weights and daily oxygenation status) that they must track and monitor trends over time in conjunction with their medical teams.

**Qualitative exemplars of theme “ongoing surveillance.”**

- “I normally take her oxygen level and if her oxygen is below 80 I take the steps and I call her doctor and check what they wanna do try the oxygen, try the nebulizer. And if not, then we end up in the emergency room most likely.” [Participant 1]
- “We’d be like, is it a bad read? Is it a good read? What’s going on? Is it the position that he’s laying in? We had to look at all the stuff. If they’d hit too hard, that would set the monitor off. If we’d go out, we had to make sure we had their O2 tanks and that their monitor was hooked up and charged and what‑not.” [Participant 2]
- “In my head. I just keep it in my head and watch her. I just watch her. I just know what to do.” [Participant 4]
- “Yeah, day in and day out, there’s not really a huge process because I am the one who’s with her every day. I’m capable of locking it in my brain. If she were to get sick though or have some kind of event, then I typically start taking notes. Basically I have a notebook, and we take note, keep track of her fluid intake or how much she’s eaten, her temperature, her mood, those kinds of things. When she’s in school, we have a notebook that goes back and forth between the school and me. If there is anything—if she was behaving a certain way that might lead them to believe that she wasn’t feeling well or couldn’t eat very much that day for whatever reason or didn’t have much of an appetite or didn’t take in too many fluids. They always let me know that so that I can be aware of it that afternoon and play detective to try and figure out if anything’s wrong. In terms of just a day to day thing, we don’t really have a schedule or a charting process.” [Participant 9]
- “Her vent, it literally keeps her alive. Because of her small lung and having all of the backup equipment too, ‘cause she does have other things that go along with the vent like an oxygen concentrator and things like that—tanks of oxygen. Being able to have all that at home means that we’re able to take care of her here and without having to rush her back to the hospital every time. Then, having it means that we also get trained in how to use it, which is great because then we’re not having to rely on nursing all the time.” [Participant 11]
- “We specifically look at her oxygen levels and then her bowel movements, which I’m sorry, I know that’s gross, but between her intestinal issues and then her breathing issues, those are the two main things that we have to keep an eye on. Usually what we’ll do is we’ll note in the planner if she’s had a bad day or some numbers seemed off.” [Participant 11]
- “Only thing that is checked every single day is his head size to make sure there’s no expansion, ‘cause that is a sign of a shunt malfunction.” [Participant 14]
- “One of the side effects of the Depakote is the gums will swell, and she’s had that consistently since she started that medicine, so she’s always got swelled gums. They come up over her teeth at times.” [Participant 20]

Numerous participants stated that they keep all the information in their heads because they do not have places to enter or monitor data. Even for caregivers who do record surveillance information, several participants expressed the magnitude of pressure for this type of ongoing assessment, as one caregiver states:

Yeah, day in and day out, there’s not really a huge process because I am the one who’s with her every day. I’m capable of locking it in my brain. If she were to get sick though or have some kind of event, then I typically start taking notes. Basically I have a notebook, and we take note, keep track of her fluid intake or how much she’s eaten, her temperature, her mood, those kinds of things. When she’s in school, we have a notebook that goes back and forth between the school and me.

Participant 9

A component of ongoing surveillance is the idea of a daily schedule that fits within the structure of the daily care plans and ongoing monitoring. The notion of a daily schedule for each child was a near-uniform experience among participants, as described in the following way:

When they’re struggling, then everyone’s struggling. Then also I think just having a plan, and having an idea of what needs to be done over the course of the day, so that you can stay on track and not lose track of time and just to fall into being complacent and destructive.

Participant 6

Ongoing surveillance was one of the process-oriented tasks that was exceedingly difficult to delegate to other members of the extended caregiving network, and many participants expressed the mental toll of constantly assessing their child with few chances for respite.

### Information Gathering

The theme of information gathering (Textbox 3) is described by caregivers as a process that often involves other members of the social network. Web-based networks are developed on social network sites, where relationships are strengthened through shared experiences, as one participant notes:

There’s a lot of camaraderie and, “Oh, my gosh, I totally forgot to clamp the G-tube at the bed last night.” A comment like that will have 50 comments: “Oh, I did the same thing the other night. Don’t worry. It’s so easy to do.”

Participant 9

Participants seemed to engage predominantly in one of several forms of information exchange: (1) information on complementary therapies that are not considered mainstream (eg, the use of cannabis products for symptom management), (2) directed advice related to troubleshooting a symptom or piece of equipment (eg, changing the button of a G-tube), or (3) engagement with long-form narrative experiences of another family with similar complexity to access information on the anticipated trajectory of illness. A participant shares interfacing with trajectory anticipatory guidance in the following way:

In a way, I’ve been able to see what our future could potentially look like, kind of an idea of the things that we’ll be up against. I am constantly, almost to a fault, I’m always researching and finding—I try to stay ahead of the next step and what’s coming next and try to be prepared, just in terms of how we’re gonna deal with it.

Participant 9

Because of the rarity of many diagnoses, or combinations of diagnoses among children with medical complexity, and the rate that new comorbidities or symptoms emerge and treatment modalities change, caregivers must engage with the information-gathering process in a dynamic and adaptive manner.

**Qualitative exemplars of the theme “information gathering.”**

- “It’s just having multiple conversations to figure out, wow, we were actually covered for that that, we could do that. In the visitations, we were given the information upfront from the NICU before we even went home. In some cases, we just floundered around, and we figured it out.” [Participant 6]
- “I ask about contraindications. I ask about side effects. I ask lots of questions and try to try to be as involved as possible. I would not consider myself a passive participant in his healthcare plan. If anything, I’m probably a little annoying because I do ask a lot of questions. I don’t just do a random Google search and look at sensational stories about terrible things that have happened to other people’s kids ‘cause I don’t think that’s either helpful or accurate. I try to go to actual trusted informational sites. I have also been known to visit the library.” [Participant 6]
- “When it comes to the seizure meds, that’s very much in collaboration with his doctor, his neurologist. We talk about what the benefits and potential side effects are and try to weigh out our options. Try to figure out the thing that’s—right now, we’re trying this new medicine.” [Participant 6]
- “I did try social networks on Facebook a lot. If I see something, I usually go to one of my groups that I’m in, and say, ‘Hey, guys, this is what’s going on. What are your opinions?’ I’ll go through and read what they have to say and things like that.” [Participant 7]
- “Because I usually get the quickest responses from them, and a lot of them are seasoned trach moms that have been through it a lot longer, things like that.” [Participant 8]
- “I had done a lotta research on CBD oil and decided to give it a try. We brought it up with our doctor. He agreed that if we thought it was best for her, that he couldn’t prescribe it. He couldn’t help us with the dosing, but if we wanted to try it, he was on board. There’s nothing he could do to stop us really. We tried it, and then not only did the muscle spasms stop, but then we saw a lot of other side effects of it that we didn’t even anticipate that were positive and very welcome. We stayed on it.” [Participant 9]
- “In a way, I’ve been able to see what our future could potentially look like, kind of an idea of the things that we’ll be up against. I am constantly, almost to a fault, I’m always researching and finding—I try to stay ahead of the next step and what’s coming next and try to be prepared, just in terms of how we’re gonna deal with it. What’s the plan? Will we need another doctor on our team? Those kind of things so a literal social network in terms of Facebook. The communities that I’m part of there are also a really big help in terms of not just planning her future but anticipating her future.” [Participant 9]
- “There’s a lot of camaraderie and, ‘Oh, my gosh, I totally forgot to clamp the G-tube at the bed last night.’ A comment like that will have 50 comments: ‘Oh, I did the same thing the other night. Don’t worry. It’s so easy to do.’” [Participant 9]

### Acute on Chronic Health Concerns

The theme acute on chronic health concerns (Textbox 4) exemplifies the cumulative intensity of the care work caregivers face when caring for children with multiple chronic conditions that experience exacerbations or new health concerns or abrupt changes from their underlying chronic conditions. Caregivers must learn to handle episodic conditions (both related to and not related to the underlying diseases), which adds to a persistent and unpredictable intensity of care work. One participant describes it in the following way:

And her immune system overall is pretty low so she catches whatever is in the air. If it’s flu season, she gets the flu. If there’s CMV season, she will get it. So it’s you know...She’s pretty much catches everything she gets in the air so it’s just kind of hard. I will say...It’s just complicated. That’s the best word to describe it. It’s complicated.

Participant 1

In this example, the child’s underlying immune system makes her more susceptible to routine and endemic childhood viruses. The reliance on technology in the home setting also makes the child more likely to experience technology challenges or malfunctions and overall changes to the therapeutic plan. This constant adaptation and vulnerability to new illnesses adds multiple layers to caregiving stress and provides a mechanism for adaptive or dynamic family management.

**Qualitative exemplars of the theme “acute on chronic health concerns.”**

- “And her immune system overall is pretty low so she catches whatever is in the air. If it’s flu season, she gets the flu. If there’s CMV season, she will get it. So it’s you know...She’s pretty much catches everything she gets in the air so it’s just kind of hard. I will say...It’s just complicated. That’s the best word to describe it. It’s complicated.” [Participant 1]
- “For about one half of the year, fall and winter, she is sick at least once a month. Anytime she gets sick, it’s always followed with bronchitis.” [Participant 3]
- “During the late fall, winter, we struggle with bugs just like any other kid. The difference between her and other typical kids, it takes her a lot longer to recover from things.” [Participant 9]

### Facilitators and Threats to Adaptive Family Management

Facilitators and threats to adaptive family management are found in Figure 1. These underlying conditions, structures, networks, and processes either aid in successful family management or provide barriers to optimal self-management practices for families of children with medical complexity and were prevalent across caregiver interviews. The facilitators and threats also highlight the networked nature of the family management experience. Family management can be influenced by the nature of the social network, intensity of caregiving activities, communication among members of the network or care team, ease of care coordination, and communication that is either present or fragmented among the health care team. Isolation because of diminished social and caregiving networks led to experiences of individual caregiver intensity and limited opportunities for information exchange. These experiences were made worse when caregivers experienced members of the clinical team who did not listen to their concerns nor make them feel as if they were a part of the team. The underlying dynamic evolution of the child’s own unique trajectory was often manifested as prognostic uncertainty, and the cumulative nature of episodic and acute exacerbations or new illnesses led to increases in stress to the caregiver and their network.

## Discussion

This qualitative study provides evidence for the development of a novel conceptual framework of adaptive family management for caregivers of children with medical complexity. The dynamic nature allows for adaptive family management processes and structures to account for changes in the child’s condition, social network composition, and underlying developmental needs and can be responsive to contextual changes in both the child and family.

There is a defined need for enhanced anticipatory guidance in the face of uncertainty in the trajectory of illness and symptom burden for caregivers of children with medical complexity. While the role of polypharmacy has been well described in populations of children with medical complexity [23], there has been less attention placed on the role of polysymptomatology or the management requirements of symptom clusters [24]. When Feudtner et al [24] assessed the symptom burden of children with medical complexity enrolled in palliative care, they noted patients had on average 12 distinct symptoms. There has been a dearth of research focused on symptom clusters in children, with much of the focus placed on children with cancer or other more singular representations of serious illness [25]. This is important because there is great uncertainty on both the side of the caregivers and the clinicians due to the great heterogeneity of presentations and lack of prognostic guidance.

Caregivers are placed in challenging positions to constantly monitor their children at home to provide evidence of even small changes from baseline condition. Genna et al [26] explored this specifically when they assessed parents’ process of recognition and response to clinical deterioration of children with medical complexity in home-based settings. They found that parents are in a constant state of vigilance because they have an intimate relationship with their children and an intuitive understanding of the nuances of their child’s condition and changes from baseline. As such, they are the clinician teams’ best supports in interval assessments when in home-based settings. This study supports these findings as well.

Due to the immense monitoring needs, complex communication strategies, approaches to documentation, financial navigation, troubleshooting technology, and management of unpleasant symptoms among a caregiving and health care clinician team social network, there is a defined role for technology-enhanced personalized approaches to home-based monitoring [27]. Previous research has recognized the inadequacy of medical devices for families of children with medical complexity, for example, the devices do not meet the family needs, are not designed to be used in locations that family requires, are organizationally disruptive, and are not designed to fit the end user [1,28]. Due to the disparities caregivers and the children in this population already experience, technology-enhanced approaches must be built alongside key stakeholders with an equity orientation to technology codevelopment [29]. The scientific community could also contribute to expanding the scientific knowledge in supporting research that can further add contextual knowledge to heterogeneous trajectories of illness and evoking personalized approaches to illness dynamics and contextual models of disease [30].

The adaptive family management framework is novel and extends previous self or family management frameworks because it highlights the networked nature of the structures, processes, facilitators, and barriers that underpin routine family management activities [8,31-33]. Additionally, it prioritizes the contextual nature of the child’s medical and developmental status. The adaptive family management framework also more purposefully highlights the role of polysymptomatology, polypharmacy, and complex symptom management needs that evolve over time and are highly variable processes [25,34]. Finally, the adaptive family management framework makes the entire social network of the family central to the understanding of how family management processes are negotiated and distributed across the network [9,35].

There are several limitations to this study that must be noted. This was a qualitative assessment, where the interviews were intended to provide depth to the contextual experiences and may lack the ability to be generalized to the population of primary caregivers who care for children with medical complexity. It was also a geographically limited sample representing a single region. As noted, the interviews took place both prior to and during the evolution of the COVID-19 pandemic, which exacerbated stresses in the context of family management during a pandemic. While we attempted to elicit the dynamic and longitudinal experience of caregivers of children with medical complexity, our interviews for this analysis were cross-sectional at a singular point in time. In the future, longitudinal interviews spanning periods of quiescence and instability or uncertainty in the care plan or between the home to hospitalized settings could more directly elucidate the challenges associated with transitions in both setting and underlying stability of the child. Further, more research is needed to pair rich narrative data with changes in the plan of care over the longitudinal perspective to understand how new changes are managed. Finally, robust social network quantitative analysis would allow us to assess the strength of bonds and stability of the social network over time. Despite those limitations, the richness of the analytical approach allows for the development of a novel framework that can be expanded upon and quantitatively operationalized for further study.

## Data Availability

The data sets generated during and analyzed during this study are available from the principal investigators (JK-M and RSV) on reasonable request.
